# 1380. Real-world Effectiveness and Complications of Valganciclovir (VGC) Prophylaxis for Kidney Transplant (KT) Recipients at High Risk for Cytomegalovirus (CMV) infection (CMV Donor (D)+/Recipient (R)-)

**DOI:** 10.1093/ofid/ofab466.1572

**Published:** 2021-12-04

**Authors:** Caroline G Roumpz, Josh Kohl, Kailey L Hughes, Amit D Raval, Yuexin Tang, Cornelius J Clancy, Minh-Hong Nguyen

**Affiliations:** 1 University of Pittsburgh, Pittsburgh, Pennsylvania; 2 University of Pittsburgh Medical Center, Pittsburgh, Pennsylvania; 3 Merck and Co., Inc., Rahway, New Jersey; 4 Merck and Co., Inc, North wales, Pennsylvania

## Abstract

**Background:**

CMV infection is common post-kidney transplant (KT). Valganciclovir (VGC) prophylaxis (Px) has lessened CMV infection among high-risk (CMV D+/R-) KT recipients (KTRs), but VGC can induce neutropenia. We quantified the burden of CMV infection among CMV D+/R- KTRs and healthcare resources required to manage these patients (pts).

**Methods:**

Retrospective study of pts undergoing KT between Jan 2014-Dec 2018. Study and control groups (gps) were CMV D+/R- and R+ KTRs, respectively. Standard post-KT immunosuppression was tacrolimus and mycophenolate mofetil (MMF). D+/R- and R+ KTRs received VGC Px (900 mg/day) for 6 and 3 months (mos), respectively.

**Results:**

Clinical characteristics did not differ between D+/R- (n=131) and R+ (n=140) pts. Median VGC Px duration was longer for D+/R- (183 vs 104 days, p< .01). Within the first 6 mos post KT, a higher proportion of D+/R- KTRs received ≥1-course of granulocyte-stimulating factor (G-CSF) (15% vs 6%, p=.02). VGC Px was stopped prematurely/intermittently in 20% and 10% of D+/R- and R+, respectively, due to neutropenia (p=0.02); corresponding data for stopping MMF for ≥1 mos were 32% and 21% (p=.05). 50% of D+/R- pts received < 3 mos Px. Leukopenia prompted hospitalization in 3% of D+/R- vs 0% of R+ pts (p=.05). CMV infections did not differ between gps (7% vs 6%, p=.80); however, VGC-resistant CMV was higher in D+/R- gp (3% vs 0%, p=.05). Between 6-12 mos post-KT, D+/R- KTRs had higher rates of CMV infection (24% vs 4%,p< .01), VGC resistance (5% vs 0%, p=.01), hospitalization due to CMV (11% vs 2%, p=.01), MD intervention (22% vs 2%,p< .01), and infectious disease (ID) referral (8% vs 2%,p= .04). 57% of CMV resistance was observed in pts who prematurely stopped VGC. Hospitalizations were longer for CMV infections in D+/R- KTRs (8 vs 1 d, p< .01). There was a trend toward higher rejection for D+/R- KTRs (13% vs 6%, p=.09).

**Conclusion:**

Universal VGC Px in D+/R- KTR remains challenging and requires significant resources for monitoring and intervention for neutropenia, including MD involvement and ID referral. Intermittent/premature stop of VGC may have led to VGC-resistant CMV,and stop of MMF may have led to a trend of higher cellular rejection at 1 yr. There is critical need for new CMV agents with a better safety profile.

**Disclosures:**

**Amit D. Raval, PhD**, **Merck and Co., Inc.** (Employee) **Yuexin Tang, PhD**, **JnJ** (Other Financial or Material Support, Spouse’s employment)**Merck & Co., Inc.** (Employee, Shareholder) **Cornelius J. Clancy, MD**, **Merck** (Grant/Research Support) **Minh-Hong Nguyen, MD**, **Merck** (Grant/Research Support)

